# Recurrent Sialolithiasis following Intraoral Deep Hilar/Intraparenchymal Stone Removal from Wharton’s Duct

**DOI:** 10.3390/jcm13030909

**Published:** 2024-02-05

**Authors:** Konstantinos Tarazis, Konstantinos Garefis, Angelos Chatziavramidis, Iordanis Konstantinidis

**Affiliations:** 2nd Academic ORL, Head and Neck Surgery Department Aristotle University of Thessaloniki, Papageorgiou Hospital, 56403 Thessaloniki, Greece; paulhawking29@gmail.com (K.T.); kgarefis@hotmail.com (K.G.); angchatziavram@hotmail.com (A.C.)

**Keywords:** sialolithiasis, recurrence, stone, Wharton’s duct, intraoral

## Abstract

(1) Background: Submandibular gland (SMG) sialolithiasis treatment has shifted significantly, favouring minimal invasiveness. Nonetheless, transoral stone removal remains viable for distal, deep hilar, and intraparenchymal stones. However, data are limited regarding recurrence and revision surgery; (2) Patients/Methods: This retrospective study included 226 patients with SMG stones treated using Wharton’s duct slitting and marsupialisation over nine years; 138 had deep hilar or intraparenchymal stones, while 88 had distal stones. Of the former group, 18 experienced symptom recurrence post-surgery, 12 with stones and 6 with duct stenosis; (3) Results: Of the 126 patients without recurrent stones, 71% were male and 29% were female. Their mean age was 51.02 ± 9.36 years. The stones of the 126 patients without recurrence had a diameter of 8.3 mm ± SD: 4 mm, which was significantly smaller than those of the patients who experienced recurrence (13.8 mm ± SD: 2.4 mm; *p* < 0.05). The mean estimated stone growth recurrence rate was 8.4 ± SD: 1.8 mm per year. A secondary operation was performed 34 ± SD: 14.7 months after the first. Of the patients with recurrence, 91.7% were treated under general anaesthesia. The preferred treatment for 58.4% of patients was intraoral revision operation; the remainder underwent total gland resection. The mean follow-up period was 43 ± SD: 18 months; (4) Conclusions: The rate of revision surgery was relatively low. In recurrent SMG sialolithiasis, new stones may grow faster than the primary stones, which are already larger than those in patients without recurrence. The slitting and marsupialisation of Wharton’s duct can treat recurrent cases.

## 1. Introduction

In recent decades, the management of submandibular gland (SMG) sialolithiasis has shifted notably, favouring minimally invasive procedures. The development of semirigid endoscopes has facilitated stone removal while preserving the gland. Compared to traditional open approaches, these methods present several advantages, including reduced morbidity and complication rates, along with significantly lower costs for the healthcare system [1]. However, the feasibility of endoscopic SMG stone removal may be limited by factors such as equipment availability, insufficient training, or a lack of reimbursement by the Greek National Health Service.

An alternative, commonly employed technique is the transoral approach. The transoral removal of SMG stones is indicated not only for distal stones but also for deep hilar and intraparenchymal stones. This procedure can be performed under local anaesthesia, primarily for distal stones; typically, it is performed under general anaesthesia for deep hilar stones [1]. The method involves incising Wharton’s duct from the ostium to the stone’s location, including the hilum, and marsupialising the submandibular duct [2].

In the current literature, SMG stone recurrence following the initial transoral removal has been reported to range from 0.5% to 19% [3,4,5,6,7,8,9,10,11]. The estimated growth rate for primary stones has been documented to be between 1 and 3 mm per year [12]. However, information on this growth rate in recurrence cases is lacking. Limited data are available; only three studies have exclusively addressed the recurrence rate after a primary duct-slitting and marsupialization procedure [2,3,6]. Finally, a specific protocol for managing recurrent SMG sialolithiasis after a primary intraoral procedure is also lacking.

The purpose of the current case series report is, firstly, to present our results concerning the management of recurrent SMG lithiasis treated primarily with an intraoral operation. Secondly, we sought to report the results of our long-term follow-up, focusing on the recurrent stone growth rate and the possibility of recurrence.

## 2. Materials and Methods

### 2.1. Patients

**Figure 1:**
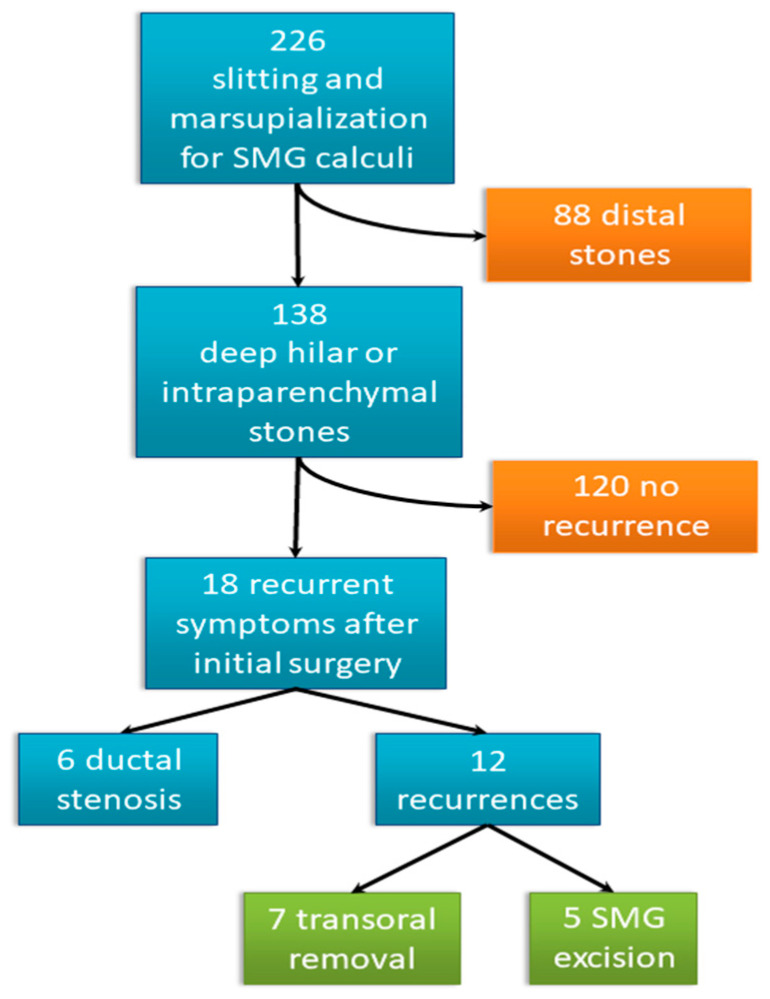
Patient flowchart.

Our retrospective study was based on the medical records of 226 patients with SMG sialolithiasis who underwent slitting and marsupialisation of their Wharton’s duct over nine years. Out of these 226 patients, 138 were diagnosed with deep hilar or intraparenchymal stones and underwent surgery, while 88 had distal stones. Eighteen of the 138 patients who underwent surgery experienced symptom recurrence after their initial procedures. Of these 18 patients, 12 had recurrent stones—recurrent stones group (RSG)—and 6 had duct stenosis (see Figure 1). The demographic data of the 126 patients with primary lithiasis with no recurrent stones (n-RSG), including their age, sex, and stone size, were recorded. For RSG, demographic data including age, sex, and disease history were recorded. Information regarding treatment type for the primary and recurrent diseases was collected. This information included the time elapsed between appearance of the first symptoms and the primary procedure, the time elapsed between the primary and secondary interventions, the stone size of the primary and recurrent diseases, the recurrent stone growth rate, postoperative complications, and the follow-up period (see Table 1). The size of the stone was determined in mm by its largest diameter, and measurements were taken after its removal.

### 2.2. Interventions

#### 2.2.1. Primary Surgical Procedure: Marsupialisation of Wharton’s Duct

**Figure 2:**
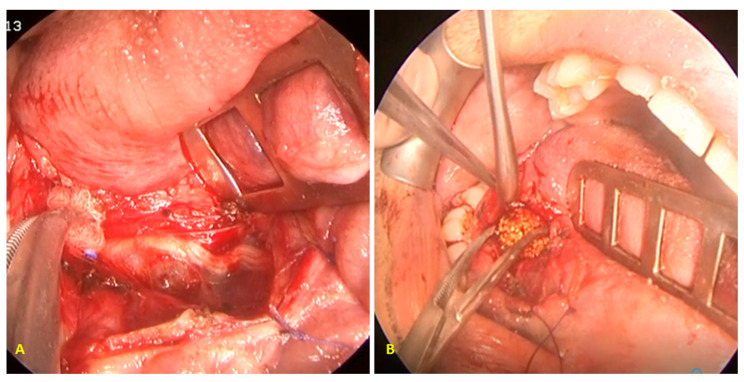
(**A**) The floor of the oral cavity was incised, and the lingual nerve was recognised and preserved. (**B**) The submandibular duct was slit, and the stone was removed using haemostatic forceps.

Currently, the Greek National Health Service does not reimburse sialendoscopy costs. Therefore, patients who were unable to cover this cost for their stone removal could choose a marsupialisation procedure (see Figure 2), as previously described, when necessary [2].

#### 2.2.2. Revision Surgery

**Figure 3:**
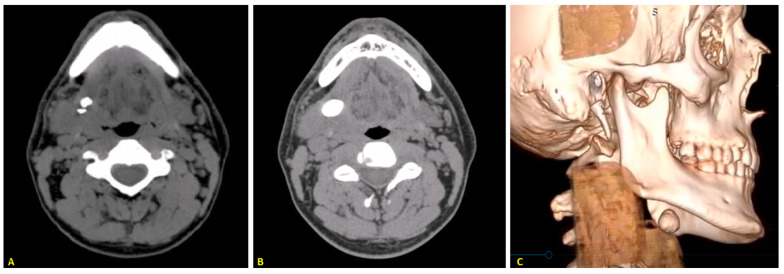
(**A**) CT scan shows the primary stone in the hilar region and a second, smaller stone located behind it. (**B**) CT showing the recurrent stone in the same patient after treatment using the slitting and marsupialisation approach. (**C**) Three-dimensional high-resolution computed tomography reconstruction showing the recurrent stone.

Patients who had experienced recurrent stones (see Figure 3) after their initial intraoral procedure were rescheduled for a secondary treatment. This treatment could involve either a secondary intraoral procedure or an open submandibulectomy.

#### 2.2.3. Secondary Intraoral Procedure

In the secondary intraoral procedure, the mucosa was carefully dissected directly at the hilar region of the affected SMG. The neo-ostium created during the previous procedure was used as a landmark. Following stone removal, the neo-ostium was enlarged as much as feasible, and the mucosa was secured with stitches to the floor of the mouth.

In all intraoral procedures, Wharton’s duct was thoroughly washed with saline after removal of the stone.

### 2.3. Follow-Up

After the initial treatments, follow-up data were collected every six months, for at least two years postoperatively, primarily through physical examinations and histories of recurrent sialadenitis. Physical examinations included palpation of the gland and its duct, as well as an assessment of the gland’s consistency, determining whether it was soft or hard. Evaluation of the quality of the saliva (clear, watery, viscus, purulent) was also performed. The history-taking process included questions about previous swelling or pain of the gland, observation of purulent saliva, or saliva with a sour/salty taste indicating sialadenitis, and the presence of spontaneous fragments of stones. For patients with suggestive histories of recurrent episodes, ultrasounds of the neck were performed. A computed tomography scan was performed in all cases of recurrent lithiasis and non-conclusive ultrasound examinations.

### 2.4. Statistical Analyses

Data analysis was performed using SPSS software (Version 28.0; IBM Corp., Armonk, NY, USA). Statistics were presented as the mean ± standard deviation or median with the range. *T*-tests and paired *t*-tests were employed to compare patient age and stone size, respectively, with a *p*-value < 0.05 considered as significant. To determine the mean growth rate of recurrent stones, we calculated the growth rate for each stone per year and then computed the mean of these rates.

## 3. Results

### 3.1. Primary Results

Of the 126 patients of n-RSG, 89 were male (71%), and 37 were female (29%). The mean patient age was 51.02 ± 9.36 years. Eighteen (13%) of these 138 patients experienced recurrent symptoms after their initial surgeries. Among them, 12 (8.6%) had recurrent stones (RSG) and 6 (4.4%) had duct stenosis. Of the patients of RSG, nine were male (75%), and three were female (25%). The mean patient age was 53.75 ± SD: 11.32 years.

No differences in age or gender were observed between the patients of RSG and the 126 patients of n-RSG (*p* < 0.05). A diagnosis of recurrent sialolithiasis was made 30.3 ± SD: 15.4 months after the primary operation, while the secondary operation was performed 4 months (range: 1–13 months) after the diagnosis and 34 ± SD: 14.7 months after the first operation, respectively. The new stones were located on the right SMG for five patients (42%) and on the left SMG for seven patients (58%), while the primary stones were found in 63 patients (46%) on the right side and 75 patients (54%) on the left side.

The mean recurrent stone diameter was 10.4 ± SD: 4.5 mm. In comparison, the mean primary stone diameter in the same patient group was 13.8 ± SD: 4 mm. However, the primary stone diameter for the remaining 126 patients with deep hilar or intraparenchymal SMG stones was 8.3 ± SD: 2.4 mm. A comparison between the primary stone size of the patients with recurrence and the recurrent stone size revealed a significant difference (*p* < 0.05): the primary stones were larger. More importantly, the primary stones of n-RSG were smaller in diameter than those of RSG (*p* < 0.05). For four patients (33%), the recurrent stone was larger than the primary stone upon diagnosis. Of the 12 patients with recurrence, 10 (83.3%) had primary stones larger than 10 mm in diameter. Of the same 12 patients, two had a secondary stone with a smaller diameter, lacking clinical significance, behind the primary stone, while the recurrent stone in one of them was larger in diameter than the primary stone. In the primary group of 138 patients, multiple stones (2–3) were identified in 21 patients in total. The mean estimated stone growth rate during recurrence was 8.4 ± SD: 1.8 mm per year. All patients who presented with recurrence—except for one such patient—were treated under general anaesthesia (11 of 12 or 91.7%). The preferred treatment method for seven patients (58.4%) was intraoral revision operation. The remaining patients (5 of 12 or 41.6%) were treated via total gland resection. The mean follow-up period was 43 ± SD: 18 months.

The data of the patients who were diagnosed with recurrence are presented in Table 1.

### 3.2. Complications after Revision Treatment

Only two complications occurred after the revision surgery. The first was stenosis of the neo-ostium in two patients (16.6%). This condition was treated with a dilation procedure under sialendoscopy. Secondly, one patient (8.3%) presented with temporary lingual nerve hypoesthesia that lasted for two weeks after the procedure. Appointments for re-evaluation after the revision surgery were scheduled, and during the follow-ups, no major complications occurred.

Of the seven patients who had undergone secondary transoral surgeries, only one (14.3%) had experienced a recurrence 42 months later. The third stone had a diameter of 3.5 mm. It was observed to be impacted at the stenosed neo-ostium, and it was removed under local anaesthesia. In cases where total gland resection was needed, we documented temporary paralysis of the marginal branch of the facial nerve in one patient (8.3%). Also, one patient (8.3%) experienced postoperative oedema in the submandibular region. It subsided after 48 h with the use of corticosteroids.

## 4. Discussion

The intraoral procedure to treat SMG sialolithiasis is well known and has been previously described [13,14,15,16,17,18]. It includes duct slitting and marsupialisation procedures [2,3,4,5,6,7,8,9,10,11,13,14,15,16,17,18,19,20,21,22,23,24,25]. However, the literature does not clarify whether a secondary intraoral procedure can be performed. To our knowledge, limited data are available on recurrent SMG sialolithiasis and its treatment.

The main findings of our study are as follows:

1. The rate of revision surgery in our patient cohort was 8.6%. This rate was relatively low, and it aligned with that reported in similar case series [2,3,4,5,6,7,8,9,10,11].
2. The mean primary stone size in patients without recurrence (8.3 mm in diameter) was significantly smaller than that in patients with recurrence (13.8 mm).
3. Secondary intraoral stone removal intervention could be an option even in revision cases (58.4%) as part of the gland preservation strategy.
4. The mean time (34 months) between the primary procedure and the secondary intervention as well as the stone growth rate (8.4 mm in diameter per year) during recurrence indicated a significant acceleration in stone formation. We have reported these findings for the first time in the literature.

First, in comparing the recurrence rate in our case series with that in the current literature, we found that approximately 8.6% of our patients had been diagnosed with a second stone during the follow-up period. In two previous studies, patients with SMG sialolithiasis were treated with the same intraoral slitting procedure, and recurrence rates between 1% and 4.6% were reported [2,3,6]. Throughout the remaining previous studies in which patients were treated with a similar transoral operation, recurrence rates of 0.5–19% were reported [3,4,5,6,7,8,9,10,11]. One review compared intraoral ductotomy with or without ductoplasty and slitting and marsupialisation techniques. Its authors concluded that the latter were associated with lower rates of distal duct stenosis postoperatively and were preferred in cases where multiple stones were present [24]. Furthermore, three studies suggested that a major contributing factor in stone recurrence is the presence of microcalculi and debris within the gland and/or the lumen of the duct after the initial approach [4,10,11]. First described in a case series by Cappacio et al. [4], the fragments of the stone and small stones left inside the duct were proposed as a probable cause of recurrence. Another study supported this notion, attributing the low recurrence rate to the postoperative use of endoscopy and dilation, which prevent symptomatic impacted stones [11]. Additionally, Galli et al. demonstrated that stone size and gland topography diameter (GTD) classification did not correlate with recurrence; instead, early recurrences were attributed to the remaining debris [10]. In both of these series, the duct was thoroughly washed after stone removal to prevent symptom reappearance, as it had been for our patients [10,26]. On the contrary, our results show that larger stones are associated with an increased recurrence rate. We hypothesise that the substantial size of recurrent stones gradually destroys the ductal system’s architecture, creating a larger and less functional ductal system. This mechanism seemed more probable in our series, as we removed the stones intraorally without leaving fragments.

Second, in individuals without recurrence, the mean size of the initial stones was 8.3 mm in diameter—significantly less than in patients who experienced recurrence (13.8 mm). Conversely, the mean primary stone size in cases with recurrence was 13.8 mm, which was significantly larger than the recurrent stones (10.4 mm). However, in 33% of patients, the recurrent stone was larger than the primary stone. Moreover, 83.3% of the patients who experienced recurrence had primary stones measuring more than 10 mm in diameter. In a retrospective cohort study by Zhang et al., 118 patients with proximal submandibular stones over 10 mm in diameter were treated similarly to our study. During the follow-up period, 14.4% experienced recurrent symptoms, with 3.4% developing newly formed stones [5]. In our series, 13% of patients had recurrent symptoms, and 8.6% developed newly formed stones. Zhang et al. suggested that stone size does not contribute to chronic obstructive symptoms or inflammation [5]. Another study demonstrated that symptoms recurred more frequently in patients with stones located in the proximal and hilar portions of the duct, which was not related to the size of the primary stone, in contrast with our data [9]. A retrospective study involving 378 patients concluded that the average stone size and stone location in the duct did not correlate with more complications, though a specific stone recurrence rate was not specified [7].

Third, our results show that a secondary transoral procedure could be an option in recurrence cases. The percentage of gland excision was low, as this procedure was employed for 3.62% of all patients. Although no specific algorithm dictating the actions to be taken in recurrence cases after intraoral procedures is available, we agree with the authors of the most current literature regarding the use of a secondary transoral approach as the preferred method in recurrent lithiasis [1,9,27]. We also propose the use of the transoral procedure in recurrence cases, especially those with easily palpable stones, to cut precisely above the stone with recognition and protection of lingual nerve, if possible. However, the transoral procedure should be avoided in excessive fibrosis cases, developed from the primary surgery, to avoid injury of the nerve and duct’s restenosis.

Finally, during our follow-up period, we observed the stones formed in patients experiencing recurrence had a remarkably accelerated growth rate of 8.4 mm in diameter per year, compared to the growth rate of 1–3 mm per year described in the literature for primary stones [12]. However, we could not directly compare the growth rate of recurrent stones with that of primary ones in our patients since we could only rely on their histories of primary symptoms, and stones may be symptomless. Recurrence was found to have occurred, on average, 30.3 months after the initial transoral removal, and a secondary operation was conducted about four months after the diagnosis and 34 months after the initial surgery.

Our study’s main limitations include its small number of recurrent sialolithiasis cases, though the main patient group was sufficient in size. Additionally, our study was retrospective, so we could not determine the growth rates of patients’ primary stones. Lastly, salivary stones have three-dimensional irregular sizes and their growth in different directions may vary; therefore, the growth rates may differ.

## 5. Conclusions

The intraoral procedure involving slitting and marsupialisation of Wharton’s duct can be used to treat recurrent SMG sialolithiasis. Its low complication and recurrence rates confirm its efficacy and safety—even in revision cases—minimising the possibility of total gland excision. The hilar location may be associated with higher relapse rates. Finally, newly formed stones might exhibit higher growth rates than primary stones in patients with recurrent disease.

## Figures and Tables

**Table 1:** Characteristics of the 12 patients with recurrence.

	Percent (%)
Sex	71% male, 29% female
Side	42% right, 58% left
Recurrent stone location	66.7% hilo-parenchymal, 8.3% hilum 25% parenchymal
Secondary intervention	58.4% intraoral, 41.6% external
	Mean (±SD)
Age (years)	53.75 (±11.32)
Primary stone size (mm)	13.8 (±4)
Recurrent stone size (mm)	10.4 (±4.5)
Time of recurrency diagnosis (months)	30.3 (±15.4)
Time between two operations (months)	34 (±14.7)
Growth rate (mm per year)	8.4 (±1.8)
Follow-up (months)	43 (±18)

## Data Availability

The data presented in this study are available upon request from the corresponding author.
